# HIV index testing services in urban Lusaka: a review of medical records

**DOI:** 10.12688/f1000research.26372.1

**Published:** 2020-09-30

**Authors:** Cibangu Katamba

**Affiliations:** 1 Lusaka Provincial Health Office, Lusaka, Zambia

**Keywords:** HIV, Index Testing, Services, Lusaka

## Abstract

**Background: **As the proportions of people living with HIV (PLHIV) who do not know their HIV infection status decrease, reaching the remaining few who are asymptomatic and not in contact with the health care system becomes a critical challenge. Therefore, reaching the first 90 of the UNAIDS 90-90-90 targets will require effective and efficient HIV testing approaches. The number of PLHIV who know their HIV status and who receive antiretroviral therapy could increase by the expansion of index testing services.

**Methods: **This project was a retrospective study looking at medical records of HIV positive clients who were elicited for index testing between October and December 2019. It was conducted in three high volume health facilities in Matero Urban sub-district 3 in Lusaka, Zambia.

**Results: **The HIV test outcomes for index contacts were as follows: 452 index contacts (53.5%) tested HIV negative, 113 index contacts (13.4%) tested HIV positive, 108 index contacts (12.8%) were known HIV positive, and 172 index contacts (20.4%) were not yet tested for HIV. Of the 113 contacts who tested HIV positive, 90 index contacts started anti-retroviral therapy within 7 days (79.6%).

The total number of 845 contacts were elicited from 604 index clients, giving a low elicitation ratio of 1:1.4. There was not much difference between gender for elicited contacts (423 men and 422 women). A total number of 565 index contacts were eligible for HIV test. 113 of them tested HIV positive, representing a positivity yield of 20%. Pearson Chi-Square test value was 0.498 and the p value was 0.481. This result is not significant since p value (0.481) is greater than the designated alpha level (0.05).

**Conclusions:** HIV programs need to explore and address barriers to HIV partner testing services to maximize HIV case identification.

## Introduction

According to the 2018 UNAIDS Global AIDS Update ^1^, there are an estimated 36.9 million people living with HIV (PLHIV). Recently, marked progress on HIV test and treat strategy has been achieved by countries’ commitment to achieve the UNAIDS 90-90-90 targets by 2020 ^1^. As of December 2017, three out of every four PLHIV knew their HIV status globally; 90% of HIV-infected individuals are expected to know their HIV status by 2020 ^1^.

According to the ZAMPHIA 2016 fact sheet ^2^, only 67.3% of PLHIV (ages 15 – 49) knew their HIV status. In 2017, Zambia had 1.1 million PLHIV and 48,000 new HIV infections ^3^. Without HIV testing services interventions targeted to key populations, including sexual partners of index clients infected with HIV, it will be hard to end the HIV epidemic by 2030 ^4^.

The cornerstone for achieving the UNAIDS 90-90-90 targets by 2020 begins with PLHIV knowing their status. As the proportions of those living with HIV who do not know their HIV infection status decrease, reaching the remaining few who are asymptomatic and not in contact with the health care system becomes a critical challenge. Therefore, reaching the first 90 goal will require effective and efficient HIV testing approaches. The number of PLHIV who know their HIV status and who receive antiretroviral therapy (ART) could increase by the expansion of index testing services. This will result in the reduction of the number of people who can transmit the virus, and subsequently in reduced new HIV infections.

The aim of this study was to review existing medical records in Zambia in order to present existing information on index testing and propose better ways to improve HIV index testing services.

## Methods

### Study design

This was a retrospective study looking at index registers of clients who tested HIV positive and were elicited for index testing between October and December 2019. The study was conducted between January and February 2020 in three high volume health facilities in Matero sub-district 3 of Lusaka district in Zambia. The study facilities included Matero First Level Hospital, Matero Main Clinic, and George Health Centre. The overview results of the study, which looked at the effectiveness of HIV index testing, were described. The analysis examined index clients’ identification, elicitations of index contacts, and testing of index contacts. The main quantitative outcome of interest for this analysis was the success of index testing to improve yield for HIV Testing Services (HTS) among female and male, and across ages among index clients; and secondly ART initiation for positive index contacts.

### Sampling

This retrospective phase of our study used a total sample enumeration technique.

The study population comprised all index clients (men and women at the study facilities) who had been diagnosed with HIV and were elicited for HIV index contact testing during the study period.

*Inclusion criteria:*

HIV positive clients (index clients or index cases) and their sexual contacts (sexual partners of index clients who have been elicited and offered HIV index testing services). The study participants included:

- HIV positive clients identified through either voluntary counseling and testing (VCT) or provider-initiated counseling and testing (PICT)
- Being documented in HIV index registers
- Having elicited at least one sexual partner

*Exclusion criteria:*

- Index clients identified through other service entry points other than VCT and PICT
- Clients not documented in index testing registers
- Contacts listed as biological children of index clients

### Data sources, variables and collection

Data on the index clients (cases) characteristics (age, gender, contacts, ART status), and the contacts’ HIV test outcome (yield, initiation status) were extracted from the HIV index testing registers into a structured pro forma.

### Data management and analysis

Data entry and analysis was performed using Statistics Package for Social Science software (SPSS version 16.0). Descriptive statistics were performed to describe the background characteristics of index clients and successful testing of index contacts. Analysis entailed simple frequencies of the main study outcomes and cross-tabulations. The association of index contacts’ gender with the HIV test outcome of the index contacts was examined using the Chi Square test.

### Ethical considerations

Ethical clearance was sought and obtained from the ERES Converge Zambian Institutional Review Board (IRB) (approval number: Ref. No. 2019-Nov-009), and authority to conduct research was obtained from the National Health Research Authority (approved on 29th January 2020) before the commencement of the study. Informed written consent for this study was waived by the IRB and National Health Research Authority due to the retrospective nature of the study. Index testing services are offered as part of the recommended national HIV testing services. Clients’ confidentiality was observed by assigning a serial number to each participant that was known only to the health care provider. Only the client’s initials and serial number appeared on the data collection forms.

## Results

The total number of index clients included in the study was 604. Matero First Level Hospital leads the participation per facility with 292 participants, followed by George Health Centre and Matero Main Clinic with 164 and 148 participants, respectively. The total number of female participants was 314 (representing 52%) and male participants was 290 (representing 48%) ( Table 1).

**Table 1.** Number of participants (index cases) by gender, month, and facility.

	Matero Main Clinic		Matero First Level Hospital		George Health Center		Total	
	Male	Female	Male	Female	Male	Female	Male	Female
**October 2019**	20	26	57	56	26	28	103	110
**November 2019**	26	28	37	25	26	28	89	81
**December 2019**	23	25	49	68	26	30	98	123
**Total**	69	79	143	149	78	86	290	314
**Grand total**	**148**		**292**		**164**		**604**	

The age of participating index clients ranged from 16 to 78 years, with mean age calculated at 34 years (SD = 9.1). Out of the total number of 604 participants, 514 clients (85.1%) were married, 85 clients (14.1%) were unmarried, 3 clients were widowed, and 2 clients were divorced.

Concerning the time spent from HIV test to the initiation of ART for index cases: 595 index clients started ART within 7 days (98.5%), 1 index client started ART within a month (0.2%), 1 index client started ART after 1 month (0.2%), and there was no evidence of starting ART for 7 clients (1.2%).

The number of contacts elicited per index client were as follows: 413 clients (68.4%) elicited 1 sexual contact each, 146 clients (24.2%) elicited 2 sexual contacts each, 40 clients (6.6%) elicited 3 sexual contacts each, and 5 clients (0.8%) elicited 4 sexual contacts each ( Table 2).

**Table 2.** Number of elicited contacts by gender, month, and facility.

	Matero Main Clinic		Matero First Level Hospital		George Health Center		Total	
	Male	Female	Male	Female	Male	Female	Male	Female
**October 2019**	30	25	113	110	35	34	178	169
**November 2019**	35	39	45	65	35	27	115	131
**December 2019**	36	35	52	55	42	32	130	122
**Total**	101	99	210	230	112	93	423	422
**Grand total**	**200**		**440**		**205**		**845**	

The mean age of elicited contacts was calculated at 33 years (range, 17–80 years SD = 9.4). From the total number of 845 elicited contacts, 604 contacts were main partners of index cases, 238 contacts were additional partners of index cases, and 3 contacts were casual.

The time spent from elicitation to HIV testing of index contacts varied across participants: 294 index contacts were tested within 7 days (34.8%), 76 index contacts were tested within 14 days (9%), 77 index contacts were tested within a month (9.1%), 133 index contacts were tested after 1 month (15.7%), and 265 index contacts were not yet tested (31.4%).

The HIV test outcomes for index contacts were as follows: 452 index contacts (53.5%) tested HIV negative, 113 index contacts (13.4%) tested HIV positive, 108 index contacts (12.8%) were known HIV positive, and 172 index contacts (20.4%) were not yet tested for HIV. Of the 113 contacts who tested HIV positive, 90 index contacts started ART within 7 days (79.6%). There was no documented evidence of starting ART for 23 HIV positive contacts (20.4%).

The total number of 565 index contacts were tested for HIV and 172 index contacts had not yet been tested for HIV ( Table 3). The Pearson Chi-Square test value was calculated at 0.498 and the p value was 0.481.

**Table 3.** Contact gender vs contact HIV status cross tabulation.

				Contact HIV status		Total
				*Tested*	*Not tested*	
**Contact** **gender**	***Male***		Count	275	89	364
			Expected Count	279.1	84.9	364.0
	***Female***		Count	290	83	373
			Expected Count	285.9	87.1	373.0
**Total**			Count	565	172	737
			Expected Count	565.0	172.0	737.0

## Discussion

Most index clients (98.5%) had documented evidence of starting ART within 7 days of HIV diagnosis. This demonstrates strongly that the test and start strategy is being implemented to scale in Matero urban sub-district of Lusaka. There was an elicitation of 845 contacts out of 604 cases, giving us a low elicitation ratio of 1:1.4. There was not much difference between gender for elicited contacts (423 males and 422 females).

A total number of 565 index contacts were eligible for HIV test. 113 of them tested HIV positive, representing a positivity yield of 20%. This index testing positivity yield is below the expected yield of above 25% as reported by several other studies ^5– 13^. The current linkage rate for positive contacts is 79.6%.

The calculated Chi-Square test value is 0.498 and the p value is 0.481. This result is not significant since p value (0.481) is greater than the designated alpha level (0.05), so we’d accept the null hypothesis that asserts the two variables are independent of each other. Therefore, there is no association between the gender of the contact and their HIV testing status.

## Conclusion

HIV index testing services are an effective way for improved HIV case identification. It has yielded a positivity rate of 20% in Matero Urban area of Lusaka. Our recommendation is that HIV programs need to explore and address barriers to HIV partner testing services to maximize HIV case finding.

## Data availability

### Underlying data

Harvard Dataverse: Cibangu, Katamba, 2020, “Replication Data for: , “HIV INDEX TESTING SERVICES IN URBAN LUSAKA: a review of medical records”, https://doi.org/10.7910/DVN/QOQM3K ^14^.

### Extended data

Harvard Dataverse: Replication Data for: HIV index testing services in urban Lusaka: a review of medical records, https://doi.org/10.7910/DVN/FSHCQ6 ^15^.

 This project contains the following underlying data:

- Data collection tool

Data are available under the terms of the Creative Commons Zero “No rights reserved” data waiver (CC0 1.0 Public domain dedication).
