# HIV index partner testing services in urban Lusaka: a retrospective review of medical records

**DOI:** 10.12688/f1000research.26372.3

**Published:** 2022-03-24

**Authors:** Cibangu Katamba

**Affiliations:** 1 Lusaka Provincial Health Office, Lusaka, Zambia

**Keywords:** HIV, Index Testing, Services, Lusaka

## Abstract

**Background: **As the proportions of people living with HIV (PLHIV) who do not know their HIV infection status decrease, reaching the remaining few who are asymptomatic and not in contact with the health care system becomes a critical challenge. Therefore, reaching the first 90 of the UNAIDS 90-90-90 targets will require effective and efficient HIV testing approaches. The number of PLHIV who know their HIV status and who receive antiretroviral therapy could increase by the expansion of index testing services.

**Methods: **This project was a retrospective study looking at medical records of HIV positive clients who were elicited for index testing between October and December 2019. It was conducted in three high volume health facilities in Matero Urban sub-district 3 in Lusaka, Zambia.

**Results: **The HIV test outcomes for index contacts were as follows: 452 index contacts (53.5%) tested HIV negative, 113 index contacts (13.4%) tested HIV positive, 108 index contacts (12.8%) were known HIV positive, and 172 index contacts (20.4%) were not yet tested for HIV. Of the 113 contacts who tested HIV positive, 90 index contacts started anti-retroviral therapy within 7 days (79.6%).

The total number of 845 contacts were elicited from 604 index clients, giving a low elicitation ratio of 1:1.4. There was not much difference between gender for elicited contacts (423 men and 422 women). A total number of 565 index contacts were eligible for HIV test. 113 of them tested HIV positive, representing a positivity yield of 20%. Pearson Chi-Square test value was 6.376 and the p value was 0.012. This result is statistically significant since p value (0.481) is smaller than the designated alpha level (0.05).

**Conclusions:** HIV programs need to explore and address barriers to HIV partner testing services to avoid over-testing and maximize HIV case identification (thus, improving HIV testing positivity yield).

## Introduction

According to the 2018 UNAIDS Global AIDS Update ^1^, there are an estimated 36.9 million people living with HIV (PLHIV). Recently, marked progress on HIV test and treat strategy has been achieved by countries’ commitment to achieve the UNAIDS 90-90-90 targets by 2020 ^1^. As of December 2017, three out of every four PLHIV knew their HIV status globally; 90% of HIV-infected individuals are expected to know their HIV status by 2020 ^1^.

According to the ZAMPHIA 2016 fact sheet ^2^, only 67.3% of PLHIV (ages 15 – 49) knew their HIV status. In 2017, Zambia had 1.1 million PLHIV and 48,000 new HIV infections ^3^. Without HIV testing services interventions targeted to key populations, including sexual partners of index clients infected with HIV, it will be hard to end the HIV epidemic by 2030 ^4^.

The cornerstone for achieving the UNAIDS 90-90-90 targets by 2020 begins with PLHIV knowing their status. As the proportions of those living with HIV who do not know their HIV infection status decrease, reaching the remaining few who are asymptomatic and not in contact with the health care system becomes a critical challenge. Therefore, reaching the first 90 goal will require effective and efficient HIV testing approaches. In Zambia, about 25% of adult men living with HIV didn’t know their HIV status in 2018. A study conducted in Zambia revealed that index testing and targeted community-based HTS are effective strategies to identify men living with HIV ^5^. Men and young people have shown limited uptake of HIV testing services under standard facility-and community-based services. Approaches such as HIV self-testing delivered at scale using several different models reached a high proportion of men, young people and first-time testers in Malawi, Zambia and Zimbabwe ^6^.

The number of PLHIV who know their HIV status and who receive antiretroviral therapy (ART) could increase by the expansion of index testing services. This will result in the reduction of the number of people who can transmit the virus, and subsequently in reduced new HIV infections. In another qualitative study conducted in Malawi and Zambia, most participants considered different approaches to partner HIV testing to be acceptable. However, there are concerns about each and implementation challenges need to be addressed ^7^.

The objective of this study was to review existing medical files and registers in Matero subdistrict of Zambia in order to describe existing information on index testing and propose better ways to improve HIV index testing positivity yield.

## Methods

### Study design

This was a retrospective study looking at index registers of clients who tested HIV positive and were elicited for index testing between October and December 2019. The study was conducted between January and February 2020 in three high volume health facilities in Matero sub-district 3 of Lusaka district in Zambia. The study facilities included Matero First Level Hospital, Matero Main Clinic, and George Health Centre. The overview results of the study, which looked at the effectiveness of HIV index testing, were described. The analysis examined index clients’ identification, elicitations of index contacts, and testing of index contacts. The main quantitative outcome of interest for this analysis was the success of index testing to improve yield for HIV Testing Services (HTS) among female and male, and across ages among index clients; and secondly ART initiation for positive index contacts.

### Sampling

This retrospective study used a total sample enumeration technique.

The study population comprised all index clients (males and females at the study facilities) who had been diagnosed with HIV, gave informed consent and were elicited for HIV index contact testing during the study period.

*Inclusion criteria:*

HIV positive clients (index clients or index cases) and their sexual contacts (sexual partners of index clients who have been elicited and offered HIV index testing services). The study participants included:

- HIV positive clients identified through either voluntary counseling and testing (VCT) or provider-initiated counseling and testing (PICT)
- Being documented in HIV index registers
- Having elicited at least one sexual partner

*Exclusion criteria:*

- Index clients identified through other service entry points other than VCT and PICT (such as MCH and VMMC)
- Index clients that accepted index testing services but did not elicit a contact
- Clients not documented in index testing registers
- Contacts listed as biological children of index clients

### Data sources, variables and collection

Data on the index clients (cases) characteristics (age, gender, contacts, ART status), and the contacts’ HIV test outcome (tested positive, tested negative, known positive, not tested, initiation status) were extracted from the HIV index testing registers into a structured pro forma. The HIV testing positivity yield was calculated (tested positive over total tested). The index testing cascade variables included: cases accepting index testing, elicitation ration, and contacts reached with testing services (disaggregated by HIV testing outcome).

### Data management and analysis

Data entry and analysis was performed using Statistics Package for Social Science software (SPSS version 16.0). Descriptive statistics were performed to describe the background characteristics of index clients and successful testing of index contacts. Analysis entailed simple frequencies of the main study outcomes and cross-tabulations. The association of index contacts’ gender with the HIV test outcome of the index contacts was examined using the Chi Square test. An additional analytical framework on index testing cascade was provided.

### Ethical considerations

Ethical clearance was sought and obtained from the ERES Converge Zambian Institutional Review Board (IRB) (approval number: Ref. No. 2019-Nov-009), and authority to conduct research was obtained from the National Health Research Authority (approved on 29th January 2020) before the commencement of the study. Informed written consent for this study was waived by the IRB and National Health Research Authority due to the retrospective nature of the study. Index testing services are offered as part of the recommended national HIV testing services. Clients’ confidentiality was observed by assigning a serial number to each participant that was known only to the health care provider. Only the client’s initials and serial number appeared on the data collection forms.

### STROBE cross sectional guidelines

We used the STROBE cross sectional reporting guidelines to ensure the study meets international standards for peer reviewed articles ^8^. A checklist was completed by entering the page numbers from the manuscript where readers can easily find each of the listed items. Where the article didn’t currently address all the items on the checklist, the text was modified to include the missing information. Where certain that an item does not apply, we wrote "n/a" and provided a short explanation.

## Results

The total number of index clients included in the study was 604. Matero First Level Hospital leads the participation per facility with 292 participants, followed by George Health Centre and Matero Main Clinic with 164 and 148 participants, respectively. The total number of female participants was 314 (representing 52%) and male participants was 290 (representing 48%) ( Table 1).

**Table 1.** Participants and their listed sexual contacts by gender, month, and facility.

	Matero Main Clinic		Matero First Level Hospital		George Health Center		Total	
	Male	Female	Male	Female	Male	Female	Male	Female
Number of participants (index cases) by gender, month, and facility								
**October 2019**	20	26	57	56	26	28	103	110
**November 2019**	26	28	37	25	26	28	89	81
**December 2019**	23	25	49	68	26	30	98	123
**Total (%)**	69 (46.6%)	79 (53.4%)	143 (49%)	149 (51%)	78 (47.6%)	86 (52.4%)	290 (48%)	314 (52%)
**Grand total**	**148**		**292**		**164**		**604**	
Number of elicited contacts by gender, month, and facility								
**October 2019**	30	25	113	110	35	34	178	169
**November 2019**	35	39	45	65	35	27	115	131
**December 2019**	36	35	52	55	42	32	130	122
**Total (%)**	101 (50.5%)	99 (49.5%)	210 (47.7%)	230 (52.3%)	112 (54.6%)	93 (45.4%)	423 (50.1%)	422 (49.9%)
**Grand total**	**200**		**440**		**205**		**845**	

The number of contacts elicited per index client were as follows: 413 clients (68.4%) elicited 1 sexual contact each, 146 clients (24.2%) elicited 2 sexual contacts each, 40 clients (6.6%) elicited 3 sexual contacts each, and 5 clients (0.8%) elicited 4 sexual contacts each.

The age of participating index clients ranged from 16 to 78 years, with mean age calculated at 34 years (SD = 9.1). Out of the total number of 604 participants, 514 clients (85.1%) were married, 85 clients (14.1%) were unmarried, 3 clients were widowed, and 2 clients were divorced as shown in Table 2.

**Table 2.** Socio-demographic characteristics of index clients and other characteristics of their referred sexual partners, Matero, Zambia, October to December 2019.

Demographic factors	Index clients (n=604)		Elicited sexual partners (n=845)	
	Number	%	Number	%
Age bands 16 to 24 25 to 34 35 to 44 45 and above	103 237 189 75	17.1 39.2 31.3 12.4	131 381 227 106	15.5 45.1 26.9 12.5
Gender Male Female	290 314	48 52	423 422	50.1 49.9
Marital status Single Married Divorced Widowed	85 514 3 2	14.1 85.1 0.5 0.3	138 704 2 1	16.3 83.3 0.2 0.1
Relationship of the elicited contact to the index client Primary or main sexual partner Additional sexual partners Casual sex			604 238 3	71.5 28.2 0.3
Testing point PITC VCT	460 144	76 24	- -	- -
Contacts testing status Tested negative Tested positive Known positive Not yet tested	- - - -	- - - -	452 113 108 172	53.5 13.4 12.8 20.4
HIV outcome status (n=565)				
Time spent from contact elicitation to HIV testing Within 7 days Within 2 weeks Within 1 month After 1 month	- - - -	- - - -	294 76 77 118	52 13.5 13.6 20.9
Tested positive Tested negative			113 452	20 80
Time spent from HIV testing to ART initiation (n = 113) Within 7 days Within 2 weeks Within 1 month After 1 month Not yet linked	- - - - -	- - - - -	89 1 0 0 23	78.8 0.8 - - 20.4

The mean age of elicited contacts was calculated at 33 years (range, 17–80 years SD = 9.4). From the total number of 845 elicited contacts, 604 contacts were main partners of index cases, 238 contacts were additional partners of index cases, and 3 contacts were casual.

Concerning the time spent from HIV test to the initiation of ART for index cases: 595 index clients started ART within 7 days (98.5%), 1 index client started ART within a month (0.2%), 1 index client started ART after 1 month (0.2%), and there was no evidence of starting ART for 7 clients (1.2%).

The time spent from elicitation to HIV testing of index contacts varied across participants: 294 index contacts were tested within 7 days (52%), 76 index contacts were tested within 14 days (13.5%), 77 index contacts were tested within a month (13.6%), 118 index contacts were tested after 1 month (20.9%). The total numbers of 565 index contacts were tested for HIV and 280 index contacts were not tested, including 108 known positive and 172 yet to be tested for HIV ( Table 2).

The HIV test outcomes for index contacts were as follows: 452 index contacts (53.5%) tested HIV negative, 113 index contacts (13.4%) tested HIV positive, 108 index contacts (12.8%) were known HIV positive, and 172 index contacts (20.4%) were not yet tested for HIV. Of the 113 contacts who tested HIV positive, 90 index contacts started ART within 7 days (79.6%). There was no documented evidence of starting ART for 23 HIV positive contacts (20.4%).

**Table 3.** HIV statuses cross tabulation by gender and age bands.

Gender of contact * HIV status Cross tabulation						Age bands *HIV status Cross tabulation					
			Contact HIV status		Total				Contact HIV status		Total
			Negative	Positive					Negative	Positive	
Gender of contact	Male	Count	232	43	275	Age band	16 – 24	Count	82	17	99
								Expected Count	79.2	19.8	99.0
		Expected Count	220.0	55.0	275.0		25 – 34	Count	199	53	252
								Expected Count	201.6	50.4	252.0
	Female	Count	220	70	290		35 – 44	Count	112	32	144
								Expected Count	115.2	28.8	144.0
		Expected Count	232.0	58.0	290.0		45≤	Count	59	11	70
								Expected Count	56.0	14.0	70.0
Total		Count	452	113	565	Total		Count	452	113	565
		Expected Count	452.0	113.0	565.0			Expected Count	452.0	113.0	565.0

The total numbers of 565 index contacts were tested for HIV. Of whom 113 tested positive and 452 tested negative (232 male and 220 female). Of the 113 people who tested positive for HIV, 43 were male and 70 female as shown in Table 3. The Pearson Chi-Square test value for the variables HIV status and gender was calculated at 6.376 (df =1) and the p value was 0. 012. The calculated Chi-Square test value for HIV status and age of index contact was 1.911 (df = 3) and the p value was 0.591.

**Figure 1.**
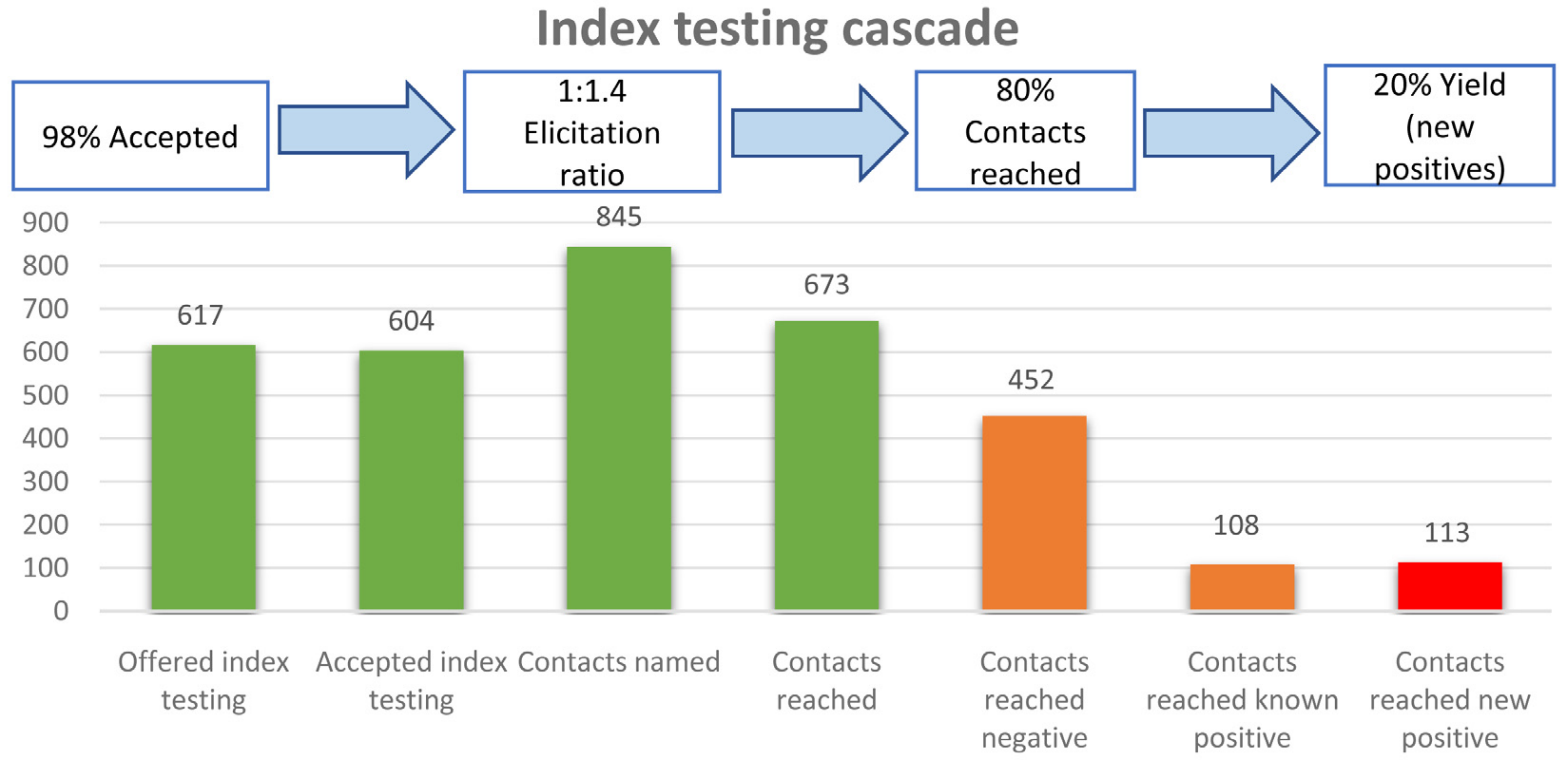
Index testing cascade.

The overall index testing cascade is represented in Figure 1 below.

The total number of HIV positive cases reported during the study period were 617, out of whom 13 did not accept index testing (had no recorded elicitation). All contacts reached accepted index testing services. Those who were already known positive were not retested for HIV. We excluded 57 cases because of elicitation of children or siblings only as contacts (without any sexual partner elicited).

## Discussion

The overall key findings of our study are shown in the result section. The 98 percent acceptability rate for index testing services in Lusaka is at an agreeable level. Though, continuous efforts are needed to cover everyone who is eligible for the service. The index cases to index contacts elicitation ratio of 1 to 1.4 is below the documented ratio of 1 to 1.8 in Zambia ^5^. There was not much difference between gender for elicited contacts (423 males and 422 females). A total number of 565 index contacts were eligible for HIV test. 113 of them tested HIV positive, representing a positivity yield of 20%. This index testing positivity yield is below the expected yield of above 25% as reported by several other studies ^9– 17^. It is not in keeping with many studies that have shown that index partner testing has the potential to increase HIV testing services (HTS) uptake; identify and diagnose HIV infected partners (yield ranging from 35% to 62% without reported intimate partner violence (IPV) ^9^. The current study has also revealed that only 80 percent of named contacts were reached with index testing services. Limitations to index testing such as relationship conflict have been documented ^7^. For partner notification, additional barriers included women losing letters, being fearful to give partners letters, being unable to read and men refusing to come to the clinic, lack of privacy or confidentiality and stigma ^7^. Other implementation challenges in personnel, resources or space have also been noted ^7^. Specific barriers to index testing in Zambia need to be explored and addressed for optimal index testing positivity yield. The current linkage rate for positive contacts is 79.6%. Most index clients (98.5%) had documented evidence of starting ART within 7 days of HIV diagnosis. This demonstrates strongly that the test and start strategy is being implemented to scale in Matero urban sub-district of Lusaka. The calculated Chi-Square test value was 6.376 and the p value was 0.012; so, there was significant association between gender and HIV status. For the variables HIV status and age, the calculated Chi-Square test value for HIV status and age of index contact was 1.911 (df = 3) and the p value was 0.591; so, there was not significant association between age of contact and HIV status.

Our study results have nonetheless provided descriptive data on the current state of index testing services in selected health facilities in Lusaka. One other strength of this study is that it can be easily reproduced elsewhere as it follows the international STROBE cross sectional study guidelines 8 . The limitation of study lies in its retrospective nature using programmatic data.

## Conclusion

HIV index testing services can be an effective way to improve HIV case identification. It has yielded a positivity rate of 20% in Matero Urban area of Lusaka. HIV positive status was not independent from gender. Further studies are needed to understand specific challenges to index testing for optimized testing yield in the context of Zambia. Our recommendation is that HIV programs need to explore and address barriers to HIV partner testing services to maximize targeted HIV case finding, minimize un-necessary testing, and ultimately improve HIV testing positivity yield.

## Data availability

### Underlying data

Harvard Dataverse: Cibangu, Katamba, 2020, “Replication Data for: , “HIV INDEX TESTING SERVICES IN URBAN LUSAKA: a review of medical records”, https://doi.org/10.7910/DVN/QOQM3K ^18^.

### Extended data

Harvard Dataverse: Replication Data for: HIV index testing services in urban Lusaka: a review of medical records, https://doi.org/10.7910/DVN/FSHCQ6 ^19^.

 This project contains the following underlying data:

- Data collection tool

Harvard Dataverse: STROBE Checklist for HIV index testing services in urban Lusaka study, https://doi.org/10.7910/DVN/SQLPBO ^20^

This project contains the following underlying data:

- STROBE-cross-sectional_checklist_Cibangu_Katamba_Index_Testing.docx

Data are available under the terms of the Creative Commons Zero “No rights reserved” data waiver (CC0 1.0 Public domain dedication).
