# Supplementary material for: HIV index partner testing services in urban Lusaka: a retrospective review of medical records
Source: F1000Res. 2022 Mar 24;9:1184. Originally published 2020 Sep 30. [Version 3] doi: 10.12688/f1000research.26372.3 (PMC9039371; doi:10.12688/f1000research.26372.3)
Supplement: Peer review history

# Peer review: Reviewer response for version 3

**DOI:** 10.5256/f1000research.122591.r128724

**Published:** 2022-04-25

**Authors:** Taurayi A. Tafuma

**Affiliations:** 1 Zimbabwe Health Interventions, Harare, Zimbabwe

The article has significantly improved. There are minor edits the author should attend to. 

1. Please cite this sentence which is in your introduction:
*"In Zambia, about 25% of adult men living with HIV didn’t know their HIV status in 2018."*
2. Results section:
  a) suggest you cancel as you are referring us to the table and this is the binary description... *" and male participants was 290 (representing 48%)"*
  b) as the above suggests deleting.. *. "3 clients were widowed, and 2 clients were divorced" *is that you are referring us to the table and these are small figures
  c) Revise this sentence. *"The total numbers of 565 index contacts were tested for HIV and 280 index contacts were not tested, including 108 known positive and 172 yet to be tested for HIV ( Table 2)."*
  d) Second last paragraph of the Results section: the first and second sentences can be deleted as it is a repetition. 
3.  Discussion: 
  a) Please rephrase this sentence as it is not clear what you mean by being agreeable. On whose basis? 
*The 98 percent acceptability rate for index testing services in Lusaka is at an **agreeable level.**
*
  b) Remove this as you are repeating the results " *There was not much difference between gender for elicited contacts (423 males and 422 females). A total number of 565 index contacts were eligible for HIV test. 113 of them tested HIV positive, representing a positivity yield of 20%." *You can add the 20% in your next sentence on this and it will still relay the message you are planning to portray e.g. The index testing positivity yield **of 20%** is below the expected yield of above 25% as reported by several other studies ^9– 17^
  c) Rephrase this. You do not need to repeat the results in this section and also check for grammatical errors *. "The calculated Chi-Square test value was 6.376 and the p-value was 0.012; so, there was a significant association between gender and HIV status. For the variables HIV status and age, the calculated Chi-Square test value for HIV status and age of index contact was 1.911 (df = 3) and the p-value was 0.591; so, there was no significant association between age of contact and HIV status."*

**If these revisions are addressed I think it will be good to proceed with indexing it.**

Is the work clearly and accurately presented and does it cite the current literature?

Partly

If applicable, is the statistical analysis and its interpretation appropriate?

No

Are all the source data underlying the results available to ensure full reproducibility?

Partly

Is the study design appropriate and is the work technically sound?

Partly

Are the conclusions drawn adequately supported by the results?

No

Are sufficient details of methods and analysis provided to allow replication by others?

No

Reviewer Expertise:

HIV, MNCH, GBV, Health Emergencies

I confirm that I have read this submission and believe that I have an appropriate level of expertise to confirm that it is of an acceptable scientific standard.

# Peer review: Reviewer response for version 2

**DOI:** 10.5256/f1000research.79223.r115749

**Published:** 2022-02-23

**Authors:** Moses Katbi, Adeoye Adegboye, Amalachukwu Ukaere

**Affiliations:** 1 U S Agency for International Development, Abuja, Nigeria; 2 Society for Family Health (SFH), Abuja, Nigeria

Safe and ethical index testing remains one of the key strategies for improving HIV case finding particularly in low prevalence communities and countries. Therefore, the study is of significant public health importance.

 The introduction is good.

 The main outcome of interest is to review the success of index testing to improve HIV case finding across gender and the different age bands. This was not shown in table 3 as the cross-tabulation compared gender and testing and not HIV status as wrongly labeled on table 3. This should be corrected. There should be a table for sociodemographic characteristics e.g., age, marital status etc. There was no age disaggregation and no cross-tabulation to compare age band and HIV status. This should be done.

 One of the inclusion criteria is having elicited at least one sexual partner, what of the index clients that accepted but did not elicit a contact, are we excluding them? If yes, include them in the exclusion list.

 The exclusion criteria includes contacts listed as biological children of index clients. Why are we excluding this group? Are we changing the title to sexual contact of index clients? If it is contact of index clients, we need to include them.

 For the tables, let us also have the percentages, you should not be quoting numbers on the tables with the narrative talking in percentages.

 There is a need to bring back all the tables with merging of some tables together. The narrative for each table should be under each table.

 Figure 1 did not talk of contact reached that refuse testing. Are there no contacts reached that refused to test?

 There is a need to review the discussion and conclusion based on findings from the new cross-tabulations and the addition of biological children if the title is to be retained.

Is the work clearly and accurately presented and does it cite the current literature?

Yes

If applicable, is the statistical analysis and its interpretation appropriate?

Partly

Are all the source data underlying the results available to ensure full reproducibility?

Yes

Is the study design appropriate and is the work technically sound?

Yes

Are the conclusions drawn adequately supported by the results?

Yes

Are sufficient details of methods and analysis provided to allow replication by others?

Yes

Reviewer Expertise:

NA

We confirm that we have read this submission and believe that we have an appropriate level of expertise to confirm that it is of an acceptable scientific standard, however we have significant reservations, as outlined above.

# Peer review: Reviewer response for version 2

**DOI:** 10.5256/f1000research.79223.r100077

**Published:** 2021-11-22

**Authors:** Soundarya Mahalingam

**Affiliations:** 1 Department of Pediatrics, Kasturba Medical College and Hospital, Manipal Academy of Higher Education, Mangalore, Karnataka, India

The revised version has been reviewed. No further changes required. Accepted for indexing.

Is the work clearly and accurately presented and does it cite the current literature?

Partly

If applicable, is the statistical analysis and its interpretation appropriate?

Yes

Are all the source data underlying the results available to ensure full reproducibility?

No

Is the study design appropriate and is the work technically sound?

Yes

Are the conclusions drawn adequately supported by the results?

Partly

Are sufficient details of methods and analysis provided to allow replication by others?

Yes

Reviewer Expertise:

Medical Education, HIV and pediatric HIV, Pediatric Allergy

I confirm that I have read this submission and believe that I have an appropriate level of expertise to confirm that it is of an acceptable scientific standard.

# Peer review: Reviewer response for version 1

**DOI:** 10.5256/f1000research.29114.r95883

**Published:** 2021-10-27

**Authors:** Taurayi A. Tafuma

**Affiliations:** 1 Zimbabwe Health Interventions, Harare, Zimbabwe

This area of study is very important at this moment where it is difficult to identify HIV positive Clients who do not know their status. However, the author needs to do a lot of literature review so that, as per the aim of this study, they can make recommendations. At this moment, the aim of the study is not in line with the findings and the analysis done. I also recommend that the title be reviewed and be improved to be in line with the narrative. 

 Introduction and Method:

- This study did not have phases so delete term phase under-sampling.
- The study population is non-specific - when do we consider someone to be a man or woman vs a boy or a girl? Best to use age categories, as this study reached out to 16-year-olds who are girls and boys.
- Clarify the exclusion criteria used, especially non-consideration of PICT and VCT index cases.
- Review the sentence on Data sources "...the contacts' HIV test outcome (yield, ...)". Is yield an outcome or do you want HIV status? Please clarify.
- The variables tracked will not provide the best ways to improve index case testing. The approach is disjointed with what the study aim is like. 

Results section:

- Being that we have a table with the results, the writer is expected to summarise the results in the narrative rather than repeat them as they are in the table.
- Generally, the author should rework this result section for a better presentation.
- Good paragraph on time spent from elicitation to HIV testing.
- The statistical powering is not clear and not sure if it is adding any value to the paper.

Discussion:

- The discussion is not elaborate enough.
- The writer also started discussing the test and start strategy - how linked is this to index case testing?
- There is no extrapolation on the findings, and this limits the assessment of these results.

Conclusion

- This is not supported by the results. Also, there seem to be no proposed ways to improve index case testing, and this could have been derived from the results. However, from the results provided so far, this paper is not in a position to provide ways to improve index case testing.

General:

- The writer needs to perform a detailed literature review for the introduction and discussion.
- There is so much literature on index case testing and this author should redo this paper

Is the work clearly and accurately presented and does it cite the current literature?

Partly

If applicable, is the statistical analysis and its interpretation appropriate?

No

Are all the source data underlying the results available to ensure full reproducibility?

Partly

Is the study design appropriate and is the work technically sound?

Partly

Are the conclusions drawn adequately supported by the results?

No

Are sufficient details of methods and analysis provided to allow replication by others?

No

Reviewer Expertise:

HIV, MNCH, GBV, Health Emergencies

I confirm that I have read this submission and believe that I have an appropriate level of expertise to state that I do not consider it to be of an acceptable scientific standard, for reasons outlined above.

# Peer review: Reviewer response for version 1

**DOI:** 10.5256/f1000research.29114.r90213

**Published:** 2021-08-03

**Authors:** Soundarya Mahalingam, Basavaprabhu Achappa

**Affiliations:** 1 Department of Pediatrics, Kasturba Medical College and Hospital, Manipal Academy of Higher Education, Mangalore, Karnataka, India; 2 Department of Medicine, Kasturba Medical College and Hospital, Manipal Academy of Higher Education, Mangalore, Karnataka, India

1. The article is a well thought out study and focuses upon the importance of contact tracing and testing in PLHIV.
2. The article needs to mention details of informed consent taken from the PLHIV while approaching their contacts for testing for HIV.
3. The study design, methods, and data collection is appropriate.
4. The details of sampling, sample size calculation, and the details of contact tracing in cases where the partners were not the only contacts needs to be elaborated upon.
5. Statistics needs to be detailed. Here the null hypothesis and the Chi square does not correlate with the aim of the study. Further statistics to assess the number of new cases identified by contact tracing and its statistical significance needs to be done.
6. Discussion needs to be rewritten. No recent references have been alluded to or compared with.
7. Conclusions mention 'HIV index testing services are an effective way for improved HIV case identification'. This statement needs to be proven appropriately with statistics.

Is the work clearly and accurately presented and does it cite the current literature?

Partly

If applicable, is the statistical analysis and its interpretation appropriate?

Yes

Are all the source data underlying the results available to ensure full reproducibility?

No

Is the study design appropriate and is the work technically sound?

Yes

Are the conclusions drawn adequately supported by the results?

Partly

Are sufficient details of methods and analysis provided to allow replication by others?

Yes

Reviewer Expertise:

Medical Education, HIV and pediatric HIV, Pediatric Allergy

We confirm that we have read this submission and believe that we have an appropriate level of expertise to confirm that it is of an acceptable scientific standard, however we have significant reservations, as outlined above.
